# Multivariate synaptic and behavioral profiling reveals new developmental endophenotypes in the prefrontal cortex

**DOI:** 10.1038/srep35504

**Published:** 2016-10-21

**Authors:** Jillian Iafrati, Arnaud Malvache, Cecilia Gonzalez Campo, M. Juliana Orejarena, Olivier Lassalle, Lamine Bouamrane, Pascale Chavis

**Affiliations:** 1 INSERM, 13009 Marseille, France; INMED UMR S 901, 13009 Marseille, France; Aix-Marseille Université, 13009 Marseille, France

## Abstract

The postnatal maturation of the prefrontal cortex (PFC) represents a period of increased vulnerability to risk factors and emergence of neuropsychiatric disorders. To disambiguate the pathophysiological mechanisms contributing to these disorders, we revisited the endophenotype approach from a developmental viewpoint. The extracellular matrix protein reelin which contributes to cellular and network plasticity, is a risk factor for several psychiatric diseases. We mapped the aggregate effect of the *RELN* risk allele on postnatal development of PFC functions by cross-sectional synaptic and behavioral analysis of reelin-haploinsufficient mice. Multivariate analysis of bootstrapped datasets revealed subgroups of phenotypic traits specific to each maturational epoch. The preeminence of synaptic AMPA/NMDA receptor content to pre-weaning and juvenile endophenotypes shifts to long-term potentiation and memory renewal during adolescence followed by NMDA-GluN2B synaptic content in adulthood. Strikingly, multivariate analysis shows that pharmacological rehabilitation of reelin haploinsufficient dysfunctions is mediated through induction of new endophenotypes rather than reversion to wild-type traits. By delineating previously unknown developmental endophenotypic sequences, we conceived a promising general strategy to disambiguate the molecular underpinnings of complex psychiatric disorders and for the rational design of pharmacotherapies in these disorders.

Prefrontal dysfunctions are associated with cognitive and executive deficits in many psychiatric diseases including schizophrenia, bipolar disorders and major depression^1^,^2^,^3^. The protracted maturation of the mammalian prefrontal cortex (PFC) into early adulthood^4^ is characterized by intense synaptogenesis, remodeling of dendritic spines and connectivity refinement in parallel to maturation of cognitive abilities^5^,^6^,^7^. Injuries occurring during this sensitive developmental period are thought to increase individuals vulnerability to psychiatric disorders^8^. Identifying the associated molecular underpinnings of this process is indispensable to fully elucidate the defective circuits underlying neuropsychiatric disorders and to rationally design innovative pharmacotherapies.

To meet this challenge, we conceived an endophenotype approach from a developmental viewpoint. Endophenotypes, defined first as internal phenotypes intermediate between genes and disease^9^,^10^, have emerged as an important method to study phenotypic variations in complex neuropsychiatric diseases^11^ and evaluate the functional consequences of risk alleles. Our goal was to identify coherent sets of multiple traits to define meaningful endophenotypes mediating neuronal dysfunctions and maladaptative behaviors during the postnatal maturation of the PFC.

To test and validate this new strategy, we chose reelin, a multifunctional protein and psychiatric risk factor. Reelin is an extracellular matrix protein expressed both in the embryonic and the postnatal brain where it exhibits multiple functions. Prenatally, reelin plays an essential role in neuronal cell migration and layer formation^12^. Postnatally, it regulates functions in several brain structures including hippocampal synaptic transmission and plasticity^13^,^14^,^15^,^16^ as well as hippocampal-dependent learning and memory^15^,^17^. Reelin is also essential in the establishment of structural, functional and behavioral properties of PFC circuits during the juvenile period^18^ and for cognitive performance at adolescent and adult stages^19^. However, the aggregate role of reelin across the different stages of postnatal maturation of the PFC remains unknown. Nonetheless, gene-association and clinical studies have revealed genetic variants in the *RELN* gene and disrupted reelin expression/signaling in a wide spectrum of psychiatric diseases (autism, schizophrenia, bipolar disorders and major depression)^20^,^21^.

To address this query, we used the reelin-haploinsufficient heterozygous reeler mouse (HRM) which recapitulates some of the molecular feature seen in these diseases^20^ and implemented a multiscale exploration in order to map structural, functional and behavioral parameters of PFC maturation throughout the first 3 months of postnatal life: before weaning (pre-weaning, Pw, P10-20), juvenile (Juv, P22-28), adolescence (Ado, P30-45) and adulthood (Adu, P50-90)^22^. To evaluate the interplay between structural, functional and behavioral phenotypes, we applied multivariate analysis to virtual mice created by data bootstrapping. Here, we show that multivariate analysis of bootstrapped morphometric, electrophysiological and behavioral datasets delineated a developmental sequence of prefrontal endophenotypes linked to the *RELN* risk allele and therefore identified neuronal targets for pharmacotherapy of reelin haploinsufficient prefrontal phenotypes. Ultimately, this strategy advances the disambiguation of complex traits markers underlying mechanisms of pharmacological rehabilitation.

## Results

### Reelin haploinsufficiency hampers morpho-functional maturation of excitatory layer 2/3-5 PrPFC synapses

**Figure 1:**
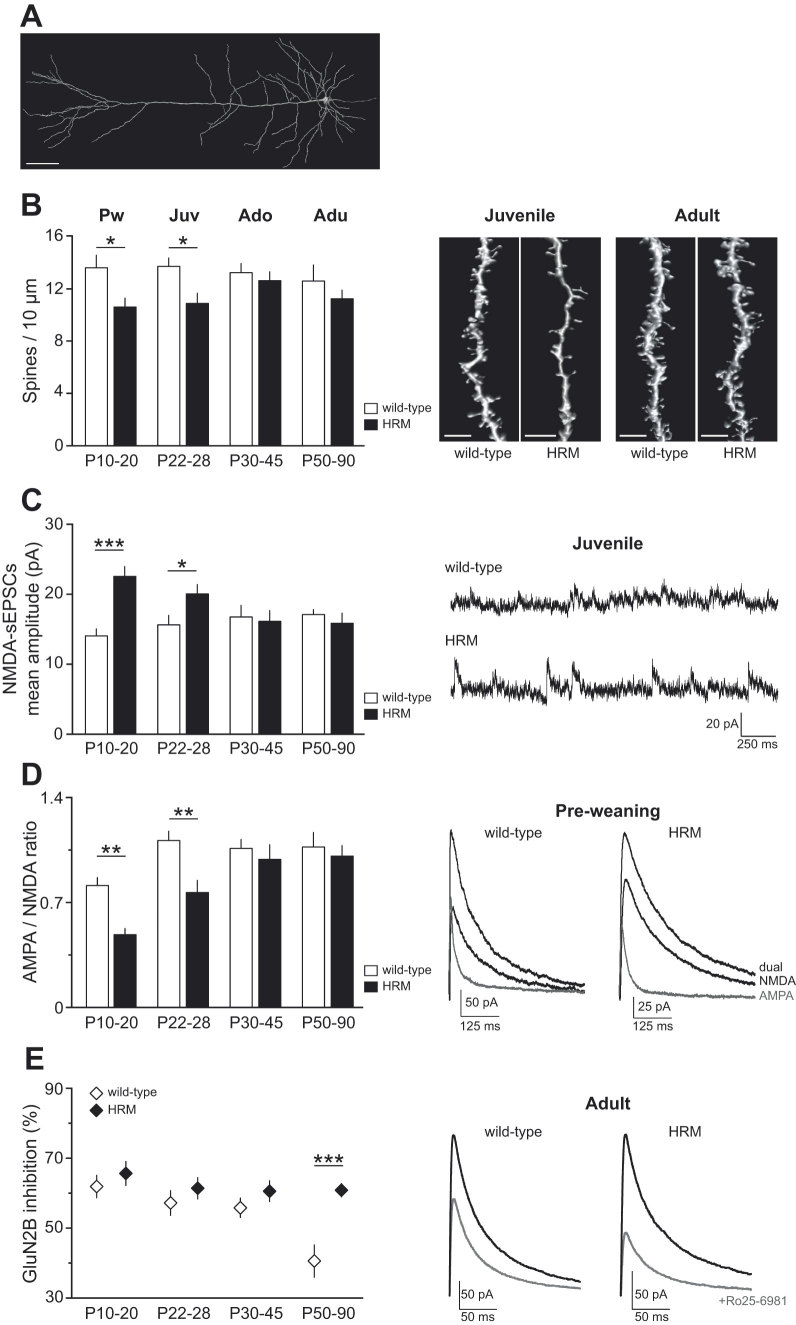
Reelin haploinsufficiency alters prefrontal dendritic spine density and synaptic glutamate receptor content. (**A**) 3D reconstructed confocal image of layer 5 pyramidal PrPFC neuron filled with neurobiotin during whole-cell recording. Calibration: 50 μm. (**B**) Average spine density per 10 μm of oblique dendritic length of layer 5 PrPFC pyramidal neuron was reduced in HRM at P10-20 (10.6 ± 0.7, n = 13 HRM versus 13.6 ± 1.0, n = 10 wild-type) and P22-28 (10.9 ± 0.8, n = 15 HRM versus 13.7 ± 0.6, n = 9 wild-type). Spine densities were equivalent between both genotypes at adolescence and adulthood. Representative 3D volume rendering of reconstructed spines and shafts. Calibration: 5 μm. (**C**) Spontaneous NMDA-EPSCs mean amplitude was significantly increased to 22.6 ± 1.4 pA (n = 14) in P10-20 HRM and to 20.1 ± 1.3 pA (n = 11) in P22-28 HRM compared to age-matched wild-type (14.1 ± 1.0 pA, n = 15, P10-20 and 15.6 ± 1.4 pA, n = 12, P22-28). Representative NMDA-sEPSCs recorded at +40 mV in the presence of DNQX. (**D**) Mean AMPA/NMDA ratio was reduced in juvenile HRM at both P10-20 (0.49 ± 0.04, n = 10 HRM versus 0.81 ± 0.05, n = 12 wild-type) and P22-28 (0.77 ± 0.08, n = 8 in HRM versus 1.11 ± 0.06, n = 8 in wild-type). No differences were observed between both genotypes at older developmental stages. Representative evoked EPSCs recorded at +40 mV in control conditions (dual EPSCs) and in the presence of NBQX (10 μM; black). AMPA-EPSCs (gray) were obtained by digital subtraction of NMDA-EPSC from the dual EPSCs. (**E**) Ro25-6981-induced inhibition of evoked NMDA-EPSCs is represented as the percent decrease in peak current in the presence of Ro25-6981 (1 μM). The percentage of Ro25-6981 inhibition is equivalent in wild-type mice and HRM between P10 and P45 (P10-20: 61.9 ± 3.2%, n = 13 wild-type and 65.7 ± 3.4%, n = 14 HRM). At the adult stage, the Ro25-6981-sensitive fraction was lower in wild-type (40.6 ± 4.6%, n = 8) compared to HRM littermates (60.9 ± 1.9%, n = 9). Representative evoked NMDA-EPSC traces at +40 mV baseline (black) and in the presence of Ro25-6981 (grey). Error bars represent SEM and n is the number of neurons. *P < 0.05, **P < 0.001 and ***P < 0.001, one-way ANOVA.

Spines and dendritic architecture are influenced by extracellular matrix^23^,^24^ and subject to intense maturational processes in the postnatal PFC^6^,^25^,^26^. We studied pyramidal neurons from deep layers (layer 5) of the prelimbic area of the medial PFC (PrPFC)^18^,^27^. In order to measure dendritic spine density and study their architecture, we performed a post hoc three-dimensional reconstruction of neurobiotin-filled deep layer PrPFC pyramidal neurons (Fig. 1A). All classes of spines were analyzed. Quantitative analysis revealed a reduction in the density of spines in HRM, restricted to the pre-weaning and juvenile periods (F_(7,72)_ = 2.409, *P* = 0.0284 ANOVA; Fig. 1B). No differences were observed between genotypes either in spine length or spine head diameter (Supplementary Table 1).

Dendritic spines are the main recipients of excitatory transmission. Thus, we examined whether these aforementioned structural abnormalities correlated with altered maturation in the synaptic content of glutamatergic ionotropic receptors. In both genotypes, the mean amplitude of AMPA-mediated spontaneous EPSCs (sEPSCs) in layer 5 pyramidal neurons remained unchanged during postnatal maturation (Supplementary Fig. 1). In contrast, the NMDA-mediated sEPSCs mean amplitude was robustly increased in pre-weaning and juvenile HRM compared to wild-type (Fig. 1C) and no differences were observed between wild-type and HRM at adolescent and adult stages (F_(7,84)_ = 5.052, *P* < 0.0001, ANOVA; Fig. 1C). When examined within each genotype, the size of NMDA-sEPSCs in HRM decreased at P30-45 and remained stable during adulthood (F_(3,40)_ = 4.785, *P* = 0.0061, ANOVA; Fig. 1C), whereas in wild-type it remained identical throughout maturation (F_(3,42)_ = 1.506, *P* = 0.2270 ANOVA; Fig. 1C).

To address whether the genotype-dependent differences in synaptic NMDA receptor (NMDAR) content reflected on the functional maturation of the synaptic gain, we compared the ratio of AMPA- to NMDA-mediated EPSCs between wild-type and HRM (Fig. 1D). The AMPA/NMDA ratio was largely reduced in P10-20 and P22-28 HRM compared to age-matched wild-type. This decrease was restricted to the pre-weaning and juvenile periods and no differences were observed between genotypes at adolescent and adult stages (F_(7,64)_ = 9.265, *P* < 0.001 ANOVA; Fig. 1D).

A hallmark feature of synaptic development in forebrain is the switch from predominantly GluN2B- to GluN2A-containing NMDARs^28^,^29^,^30^, which is reflected by a reduced sensitivity of NMDA-EPSC towards allosteric modulators of GluN1/GluN2B^31^,^32^,^33^. We previously showed that decreasing reelin levels or impairing reelin signaling delayed the maturational switch of NMDARs from GluN2B- to GluN2A-containing receptors in the hippocampus^14^,^16^,^34^. To examine the impact of reelin haploinsufficiency in the subunit composition of NMDAR across PrPFC development, we characterized the maturational time course of NMDA-EPSC inhibition by the noncompetitive GluN2B-selective antagonist Ro25-6981 (Fig. 1E). In wild-type mice, the fraction of Ro25-6981-sensitive NMDA-EPSCs remained elevated from pre-weaning to adolescent period and decreased abruptly during adulthood (F_(3,32)_ = 6.603, *P* = 0.0013, ANOVA; Fig. 1E), reflecting a reduction in GluN2B-NMDARs in adult PrPFC. In pre-weaning, juvenile and adolescent HRM, the Ro25-6981 inhibition of NMDA-EPSCs was similar to age-matched wild-type. However in adult HRM, the effect of Ro25-6981 was significantly larger compared to wild-type mice, showing that the proportion of GluN2B-NMDARs remains elevated in HRM at this stage (F_(7,69)_ = 4.826, *P* = 0.0002, ANOVA; Fig. 1E). Thus in the PrPFC, the classical developmental switch in the subunit composition of synaptic NMDARs occurs much later that in other forebrain structures^31^. Consistent with our previous studies^14^,^16^, reelin haploinsufficiency prevented NMDAR subunit switching normally observed in adult PrPFC deep layer pyramidal neurons.

### Reelin haploinsufficiency uncovers a correlation between disrupted LTP and fear memory erasure

**Figure 2:**
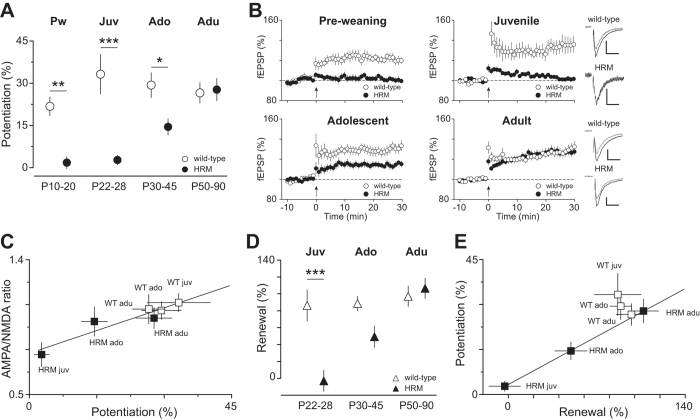
Reelin haploinsufficiency disrupts long-term potentiation and renewal of associative learning. (**A**) Maturational profile of LTP. No potentiation was observed in HRM between P10-20 and P22-28 compared to age-matched wild-type (P10-20: 1.7 ± 2.1%, n = 12 HRM versus 21.8 ± 3.3% n = 8 wild-type; P22-28: 2.7 ± 1.7%, n = 18 HRM versus 33.2 ± 7.0%, n = 10 wild-type). LTP gradually increased in P30-45 HRM (14.5 ± 2.9%, n = 13) to reach values similar to wild-type at P50-90 (27.8 ± 4.0%, n = 16 HRM and 26.6 ± 3.6%, n = 18 wild-type). (**B**) Grouped time courses of fEPSP responses expressed as percentage of baseline before and after plasticity induction (indicated by arrow) in HRM and wild-type littermates are depicted for each developmental period. Right: Representative traces averaged from ten fEPSP responses before (gray) and 30 min after plasticity induction (black) taken from juvenile and adult mice. Calibration: 0.1 mV, 10 ms. (**C)** The coefficient of correlation between AMPA/NMDA ratio and the percentage of LTP is r^2^ = 0.8871 (*P* = 0.005) showing that the maturational changes in AMPA/NMDA ratio are tightly correlated to the degree of LTP. (**D**) Percentage of context-dependent renewal of fear response plotted against the age at which mice were conditioned. Renewal was similar at all ages in wild-type mice (Juv: 86.4 ± 18.5%, n = 15; Ado: 88.7 ± 8.6, n = 14; Adu: 97.1 ± 12.0%, n = 17) whereas in HRM, it gradually increased from -2.8 ± 12.4% (n = 21) in juvenile to 49.4 ± 12.7% (n = 16) in adolescent and reached values similar to wild-type at adult stage (106.6 ± 12.0%, n = 17). Data are the average of freezing response to the first 2 CS presentations during renewal normalized to the average of the last 2 CS presentations (CS 23-24) during extinction. (**E**) The correlation between percentage of renewal and magnitude of LTP during maturation is described by a linear fit with a slope of 0.9999 for HRM. Error bars represent SEM and n is the number of animals. *P < 0.05, **P < 0.001 and ***P < 0.001, one-way ANOVA.

Alterations in spine density and/or structural and basic synaptic properties are likely to impact on synaptic plasticity. In wild-type mice, theta-burst stimulation induced a robust long-term potentiation (LTP) of field excitatory postsynaptic potentials (fEPSP) measured at synapses onto layer 5 pyramidal neurons, the magnitude of which remained similar in all age groups (F_(3,43)_ = 0.8102, *P* = 0.4952 ANOVA; Fig. 2A,B). LTP was blocked by the NMDAR antagonist AP5, thereby confirming mediation by NMDARs (not shown)^35^. In marked contrast, LTP was absent in HRM throughout the pre-weaning and juvenile period, reduced during adolescence and robustly expressed during adulthood (F_(3,55)_ = 19.08, *P* < 0.001 ANOVA; Fig. 2A,B). The magnitude of LTP was highly correlated to the AMPA/NMDA ratio in the maturing PrPFC (Fig. 2C), further validating this ratio as a reliable indicator of synaptic plasticity. Together, these data reveal that normal levels of reelin are necessary for the expression of NMDAR-dependent LTP in the PrPFC during early postnatal development.

To determine whether reelin haploinsufficiency disrupts higher congnitive functions, we tested the long-term renewal of fear memory acquired during fear conditioning, a classical form of associative learning^36^. Mice were trained from the juvenile period to adulthood. No differences were observed between wild-type and HRM from P22 to P90, either in the associative conditioned learning or in the extinction of learned fear behavior (not shown). Thus, acquisition and extinction of associative fear memories are not affected by reelin haploinsufficiency. To test the renewal of the original memory, mice were re-exposed to the conditioned stimulus in the acquisition context 7 days after extinction^18^. Wild-type mice exhibited a stable context-dependent renewal of fear memory from the juvenile age to adulthood (F_(2,44)_ = 1.194, *P* = 0.3126 ANOVA; Fig. 2D). In contrast, renewal was impaired in HRM trained during the juvenile period, gradually increased during adolescence and became identical to wild-type at adulthood (F_(5,94)_  = 11.38, *P* < 0.0001, ANOVA; Fig. 2D). Notably, the maturational profiles of renewal and LTP were tightly correlated (Fig. 2E).

Thus, classical variance analysis of single parameters measured across time x genotype and regression analysis between two parameters, showed that during postnatal PrPFC development, reelin controls the covariation of multiple parameters such as the synaptic GluR content, synaptic gain and plasticity as well as renewal of memory.

### Computation of virtual mice

The classical univariate analysis thus far employed precludes the consideration of the multidimensional nature of the data and evaluation of the interrelationships between structural, functional and behavioral dysfunctions. To address these issues, we next proceeded to a global analysis and used multivariate analysis of variance (MANOVA) which allows a robust comparison of wild-type mice and HRM across maturation. However, MANOVA requires all parameters to be measured in the same animal. Since this is not feasible due to experimental constraints, we created virtual mice (Supplementary Figures 2 and 3). For this purpose, we computed bootstrapped datasets (e.g. virtual samples) by using a parametric (normal) bootstrap method to create random samples based on experimental data distributions obtained for each parameter (Supplementary Figure 2).

Prior to bootstrapping, we tested the deviation from normality of each parameter by comparing the t-statistic of the sample to the “95% rule” of normal distributions: 52 out of 59 samples (parameter x age x genotype) displayed less than 5% “2σ events”, the 7 remaining samples (spread among 5 parameters) had only one event bigger than 2σ (likely experimental outliers) but the percentage was bigger than 5% due to small number of points in the sample (Table 1). Distributions were then built from experimental data for both genotypes in each maturational period using a normal distribution for each parameter. A robust estimate of the probability distribution function (PDF) was computed using the following equation: 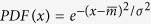. The Gaussian function was centered at the median value 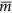 and we used the interquartile range (IQR) of the data as an estimation of the standard deviation σ = IRQ/1.35 (the value 1.35 corresponds to the IQR of a normal distribution with a standard deviation equaled to 1). We then randomly created a large set of virtual samples (n = 100,000 per group) following experimentally-deduced distributions for each group (age and genotype) (Supplementary Figures 2 and 3).

**Figure 3:**
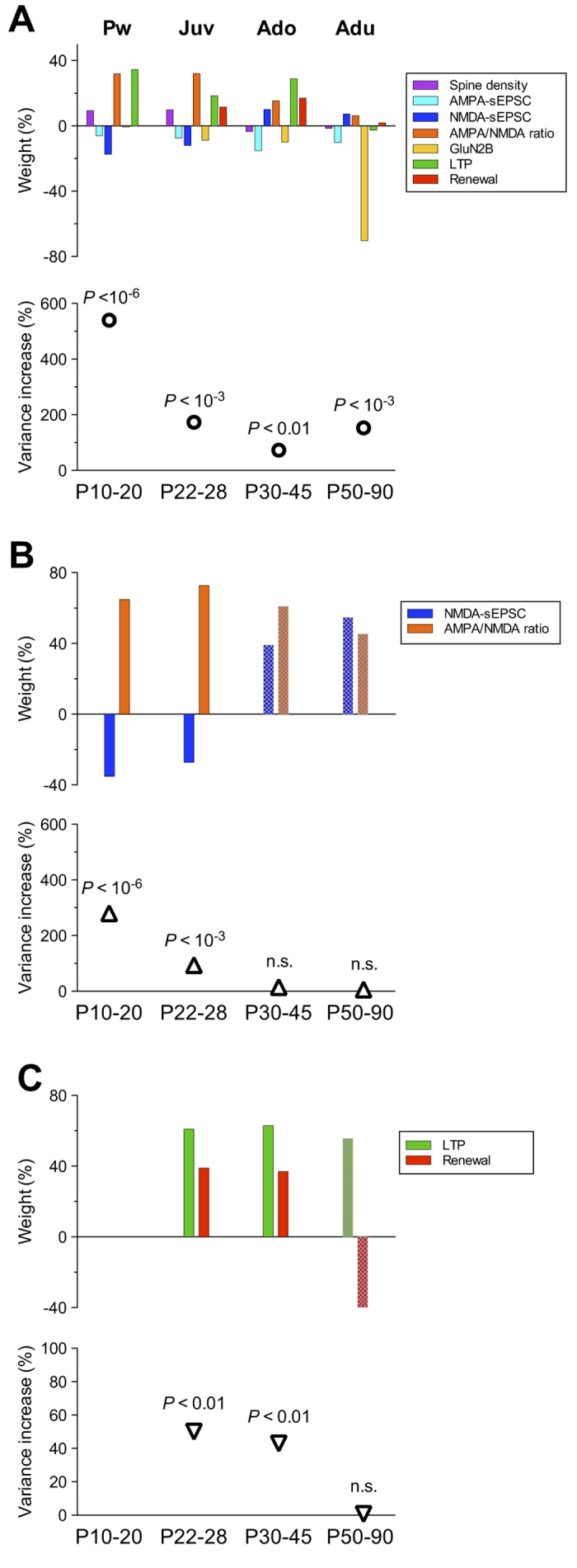
Maturational profile of population differences based on optimal parameters combination. (**A**) The relative weight of measured parameters - spine density, AMPA- and NMDA-sEPSCs amplitude, AMPA/NMDA ratio, Ro25-6981 sensitivity of evoked NMDA-EPSCs (GluN2B), % of LTP and renewal - is shown within each developmental period. Note that the weights reflect the magnitude (absolute value) and direction (sign) of the differences between distributions showed in Supplementary Figure 3. The increase in the variance and the median *P* values of the optimized parameter was 540% and 4.1 × 10^−9^ at P10-20, 173% and 2.3 × 10^−5^ at P22-28, 73% and 1.1 × 10^−3^ at P30-45, 153% and 2.3 × 10^−5^ at P50-90. (**B**) Relative weight of NMDA-sEPSCs amplitude and AMPA/NMDA ratio during postnatal maturation. The variance increase of the optimized parameter combining the 2 parameters was strongly reduced from 280% at P10-20, 94% at P22-28 to 15% and 6% at P30-45 and P50-90. The median *P* values were 6 × 10^−7^ at P10-20, 4 × 10^−4^ at P22-28, 0.1 at P30-45 and 0.26 at P50-90. (**C**) Developmental time course of the relative weight of LTP and renewal. The variance increase of the optimized parameter combining LTP and renewal was 50% at P22-28, 43% at P30-45 and 0.7% at P50-90. Median *P* values were 5 × 10^−3^ at P22-28, 8 × 10^−3^ at P30-45 and 0.46 at P50-90. Shaded bars indicate when the variance increase of the optimized parameter does not reach significance.

Then, the MANOVA was computed between wild-type mice and HRM within each developmental period. This analysis provided the relative contribution of each parameter. Derived from the weight of each parameter, an optimized weighted parameter was calculated to maximize the difference between both genotypes at each developmental stage and its robustness was quantified by the variance increase of MANOVA (Fig. 3). Finally, the optimized parameters were used to estimate the statistical significance of the difference between wild-type mice and HRM (Fig. 3). Based on the average number of experimental points obtained for each group, we created 10 random virtual samples per group, age x genotype. To obtain a median *P* value for each time period, a t-test on the distributions of the optimized parameters between both genotypes was iterated 1000 times.

### Multivariate analysis reveals subgroups of phenotypic traits specific of prefrontal maturational epochs

We applied MANOVA to bootstrapped datasets and statistically analyzed the distribution pattern of all parameters measured in both genotypes during maturation as well as their global contribution to the difference between the two genotypes (Fig. 3). We found that the relative weight of each parameter varied across the different developmental periods (Fig. 3A top). Analysis of the relative weight revealed a predominant contribution of NMDA-sEPSCs, AMPA/NMDA ratio and LTP during the pre-weaning and juvenile periods (P10-20 and P22-28; Fig. 3A top). At the adolescent stage, LTP and magnitude of renewal exhibited the most elevated weight, which shifted to GluN2B-containing NMDARs at the adult stage. The weighted contribution of spine density was overall weak across maturation.

We next calculated a global parameter that provides an optimized combination of measured factors: spine density, AMPA- and NMDA-sEPSCs amplitudes, AMPA/NMDA ratio, Ro25-6981-sensitivty of NMDA-EPSCs (GluN2B), LTP and magnitude of renewal, thereby permitting an optimal estimation of the statistical difference between wild-type and HRM across multiple postnatal maturation points. The median *P* value of the optimized parameter combining all parameters revealed that the two genotypes are statistically different at all maturational stages (Fig. 3A bottom). The variance increase of the MANOVA was the highest during the pre-weaning period and was reduced starting from the juvenile period. The increase in the variance and the median *P* value of the optimized parameter were lower during adolescence compared to juvenile and adult periods. Notably, they were similar between juvenile and adults showing that HRM and wild-type mice are equally different during these two developmental periods.

To further test the robustness of our approach, we applied MANOVA on the parameters that were not different between genotypes when using single parameter analysis (Figs 1B–E and 2D) at P30-45, the developmental period for which the MANOVA revealed a lower difference between wild-type mice and HRM (Supplementary Figure 4). Here, we found that the variance increase of the optimized parameter was 42% and that the two genotypes became statistically different (*P* < 0.01; Supplementary Figure 4). These data show that multivariate analysis is very powerful as it takes into account the aggregate effect of multiple factors, thus revealing significance that was masked when parameters were considered independently.

We next examined whether the difference between wild-type mice and HRM resulted from parameter interactions specific to each maturational period. The combination of NMDA-sEPSC amplitude and AMPA/NMDA ratio accounted for more than half of the variance increase of all parameters from P10 to P28 (52% at P10-20 and 54% at P22-28) and became undistinguishable between wild-type and HRM from P30 to P90 (Fig. 3B), showing they predominantly contribute to the difference between both genotypes during the pre-weaning and juvenile periods. We then analyzed the combination LTP and renewal and found that their relative weights were similar at P22-28 and P30-45. The variance increase of the resulting parameter represented 59% of the variance increase of all parameters at P30-45 (Fig. 3C), showing that during adolescence the difference between genotypes weighed primarily on LTP and fear renewal. During adulthood, the proportion of GluN2B-containing NMDA receptors sustained the difference between both genotypes (Fig. 3A).

Thus, multivariate analysis of bootstrapped data strengthens and extends further our previous conclusions based on single parameter analysis. In particular, this approach illuminates statistical differences between wild-type mice and HRM from the pre-weaning to the adult period. Furthermore, this approach allowed the establishment of the precise contribution of individual parameters to these differences. Altogether, these data revealed developmental distribution patterns of assorted parameters, thus delineating endophenotypes, linked to the susceptibility *RELN* gene, specific to each prefrontal maturational epoch.

### Ketamine treatment in juvenile HRM restores synaptic and behavioral functions but does not recapitulate wild-type endophenotype

Thus far, we have identified population parameters and developmental windows at which they predominantly contribute to prefrontal endophenotypes. Considering the predominance of NMDARs to juvenile endophenotype (Fig. 3B), we hypothesized that targeting NMDARs could repair dysfunctional PFC in reelin-haploinsufficient mice and examined the endophenotypic effect of the NMDAR antagonist ketamine administered to juvenile HRM^18^.

**Figure 4:**
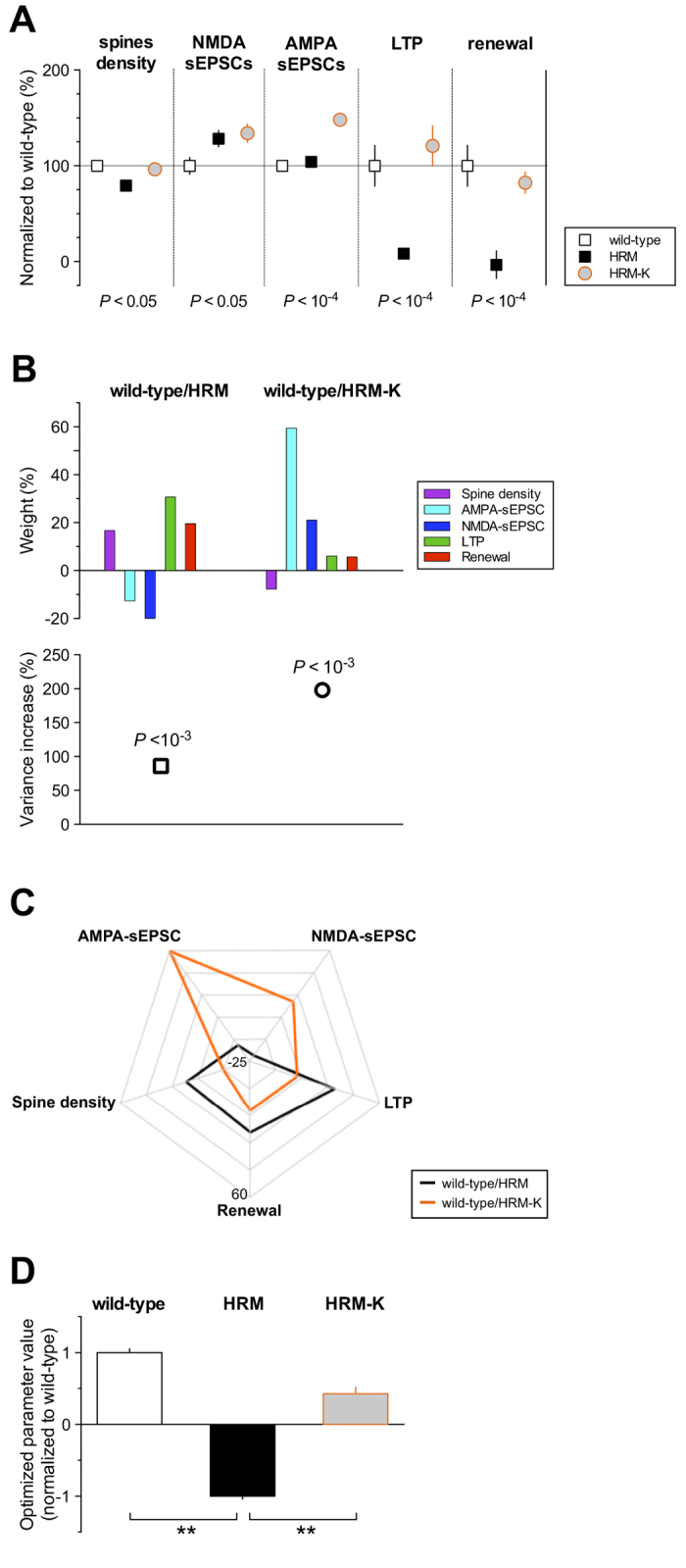
Effect of the NMDAR antagonist ketamine on the multivariate profile of juvenile reelin-haploinsufficient mice. (**A**) Summary graph of multiscale measurements performed at P22-28 in wild-type, HRM and HRM injected with ketamine (HRM-K, 30-100 mg/kg). Each group of measure is expressed as a percentage ± SEM of the mean value obtained in wild-type mice. For each parameter, *P* values obtained with one-way ANOVA are indicated. Multiple comparisons showed that spine density, LTP and renewal were not different between wild-type and HRM-K, whereas NMDA-sEPSCs and AMPA-sEPSCs amplitudes were different in HRM treated with ketamine compared to wild-type mice. Note that measures presented for HRM-K are taken from datasets in ref 18. (**B**) The relative weight of each parameter is showed for wild-type versus HRM untreated (HRM) or versus ketamine-treated HRM (HRM-K). The increase in the variance and the median *P* values of the optimized parameter were: 86% and 6.5 × 10^−4^ for wild-type/HRM, 198% and 1.4 × 10^−5^ for wild-type/HRM-K. (**C**) Radar chart showing the divergence of relative weights (%) between wild-type versus HRM and wild-type versus ketamine-treated HRM. (**D**) Bar graph showing the optimized parameter value ± SD for each genotype and treatment normalized to the value obtained for wild-type mice. Optimized parameter value for each condition computed by applying the relative weights obtained from the MANOVA in untreated condition (b, upper left) on the multiscale measurements obtained for each genotype and treatment. The optimized parameter value was not different between wild-type and HRM injected with ketamine (HRM-K; *P* = 0.27, Student’s t-test). Wild-type and HRM-K optimized parameters were significantly different from HRM (*P* = 6.5 × 10^−4^ and *P* = 2.3 × 10^−3^ respectively, Student’s t-test).

In support of this hypothesis, we recently found that a single *in vivo* injection of ketamine reinstated spine density, LTP and fear renewal in HRM and markedly enhanced AMPA-sEPSC amplitude without affecting NMDA-sEPSC amplitude^18^ (Fig. 4A). Using MANOVA, we analyzed the distribution pattern of these parameters in P22-28 wild-type mice and HRM either untreated or injected with ketamine^18^ (Fig. 4B). We first verified that the variance increase of the MANOVA for these 5 parameters still sustained the statistical difference between both genotypes in untreated conditions (wild-type/HRM, *P* = 6.5 × 10^−4^; Fig. 4B). To evaluate the robustness of the rehabilitation by ketamine, we used the weights obtained from the MANOVA to compute a global parameter combining these 5 parameters (Fig. 4D). The global parameter for ketamine-treated HRM was not different from wild-type (*P* = 0.27, Student’s t-test), showing that ketamine is effective in globally rescuing reelin-haploinsufficient dependent deficits in juvenile PrPFC (Fig. 4D).

Finally, we dissected the pattern of parameters distribution underpinning ketamine rehabilitation and analyzed their relative weight by comparing wild-type to HRM injected with ketamine (wild-type/HRM-K; Fig. 4B). The variance increase in the MANOVA revealed that ketamine treatment induced a pattern of parameters distribution significantly different from the one obtained in wild-type (wild-type/HRM-K, *P* = 1.4 × 10^−5^; Fig. 4B), showing that HRM-K do not recapitulate wild-type juvenile phenotypic traits. The relative weight analysis illustrates that this results from a switch in factors contributing predominantly to the difference between wild-type and HRM in different condition, e.g. from LTP and NMDA-sEPSCs in untreated to AMPA-sEPSCs in ketamine-injected mice (Fig. 4B,C).

Together, these results demonstrate that ketamine administration at the juvenile stage robustly rehabilitated prefrontal properties through induction of new endophenotype rather than a reversal to wild-type phenotypic traits.

## Discussion

By combining cross-sectional and multiscale phenotypical exploration with the generation of virtual mice to allow multivariate statistical evaluation, we devised a new strategy to analyze the interplay between the multiple variables of morphology, function, behavior and age. We also validated this approach as a useful tool to identify new developmental endophenotypes and predict neuronal targets and therapeutic windows.

In wild-type mice, parameters directly related with basic properties of excitatory synapses - spine density, AMPA- and NMDA-sEPSCs amplitude, magnitude of LTP - did not significantly change from the pre-weaning juvenile period to 3 months of age (adulthood), showing that the layer 2/3 to layer 5 excitatory projections exhibit robust early functionality. In contrast, at hippocampal CA1 synapses, the AMPA/NMDA ratio and LTP increase from pre-weaning (P14) to adolescence (6–7 weeks)^37^, as does the AMPA/NMDA ratio at nucleus accumbens synapses between juvenile and adult stages^38^, reinforcing the peculiarity of postnatal PFC maturation. We also found, in agreement with a previous study^31^, that the decrease in synaptic GluN2B-containing NMDARs is delayed towards adulthood. This is in contrast to other structures - hippocampal CA1 pyramidal cells, medium spiny neurons of the nucleus accumbens and pyramidal cells of the barrel cortex - in which the decreased sensitivity to GluN2B antagonist is completed as early as the second postnatal week^28^,^33^,^38^,^39^,^40^. The remaining fraction of GluN2B antagonist-insensitive current is similar either in PrPFC, hippocampus or accumbens suggesting that the proportion of GluN2B-NMDAR is equivalent in these limbic structures^33^,^38^,^40^. Interestingly, these structures undergo incomplete GluN2B subunit switching compared to the barrel cortex^39^,^40^.

In adult HRM, the NMDAR subunit composition is kept in an immature stage characterized by an increased sensitivity to the GluN2B-NMDAR selective antagonist Ro25-6981. This finding reinforces our previous studies which showed that, *in vitro*, reelin is necessary for the maturation of NMDARs^14^,^16^,^34^. It is acknowledged that the developmental switch from primarily GluN2B- to predominant GluN2A-containing NMDARs occurs at a time window coincident with synapses maturation, circuit refinement and acquisition of learning abilities^41^. Thus, the elevated contribution of GluN2B in the adult PrPFC of HRM could be an aggravating factor for prefrontal dysfunction in psychiatric disorders related to reelin haploinsufficiency.

Using traditional univariate analysis, we showed that in pre-weaning and juvenile HRM, reduction of spine density is concomitant to a decreased AMPA/NMDA ratio, the latter resulting from an increased amplitude of NMDA-sEPSCs. In juvenile and adolescent HRM, disruption of LTP is age-correlated to deficits in fear renewal. At adult stages, spine density, stoichiometry of synaptic glutamatergic receptors, long-term plasticity and memory are recovered to normal levels in HRM. Similarly, it has been shown that adult HRM exhibit very minimal hippocampal spine density deficits compared to wild-type^42^. These observations could be explained by the existence of homeostatic processes or adaptative mechanisms engaging different signal transduction pathways at the adult stage. However, it does not exclude that deficiencies observed at pre-weaning and juvenile stages will affect adult properties differently than those measured in this study or will render adult HRM more susceptible to mild environmental insults^43^,^44^.

Most behavioral reports on adult HRM gave conflicting results with studies showing that HRM exhibit normal responses in a wide range of behavioral measures^45^,^46^ while other showed abnormal behavioral responses^15^,^47^,^48^. This lack of consensus challenged the validity of a role of reelin dysfunctions in the etiology of psychiatric disorders. Here, we showed that HRM exhibit a reduction in the long-term retention of associative fear-conditioned memory from the juvenile period to adolescence, a behavior which became similar to wild-type during adulthood, thus extending further our previous report^18^. Despite this return to normal behavior at the adult stage, MANOVA showed that the difference between adults wild-type and HRM display the same level of statistical significance as juveniles.

To gain a more sensitive and powerful insight in our cross-sectional study and in the analysis of multiple traits of PFC postnatal maturation, we created virtual mice based on the distribution of experimental data and performed a multivariate analysis of bootstrapped datasets. For the first time, our results show that wild-type mice and reelin haploinsufficient mice are significantly different during the pre-weaning period through adulthood and that the differences between both genotypes resulted from parameter interactions specific to each maturational period and not from a simple developmental delay. By dissecting the predominant contributors to this difference, we found that synaptic glutamate receptor content prevailed at pre-weaning and juvenile stages, while synaptic plasticity and memory retention are increasing contributors at adolescence, which are superseded by synaptic NMDAR subtype in adulthood. Spine density scarcely contributed to the difference between both genotypes at any ages, thus challenging the paramount role attributed to abnormal spine density and/or morphology in underlying cognitive deficits occurring in psychiatric diseases^49^,^50^,^51^. Altogether, our data show that properties of prefrontal excitatory layer 5 synapses represent a complex trait marker that may be considered as a reliable endophenotype.

We quantified the precise contribution of multiscale parameters to prefrontal endophenotypes linked to reelin-haploinsufficiency in order to understand the mechanisms linked to dysfunctions and propose therapies. We found this strategy was very efficient as it revealed not only the molecular substrate and type of pharmacological compound but also the appropriate maturational period. We previously showed that a single injection of ketamine, an anesthetic and fast acting antidepressant^52^,^53^, during the juvenile period induced a pharmacological restoration of reelin-haploinsufficient mice^18^. Here, we confirmed the robustness of ketamine rehabilitation and showed that ketamine treatment changed the distribution pattern of multiscale parameters demonstrating that the endophenotype of pharmacologically rehabilitated HRM differs from wild-type animals.

In conclusion, we report here that the analysis of interrelationships between multiple variables - structure, function, behavior and age, allows the identification of new developmental endophenotypes and prediction of neuronal targets for pharmacotherapy. The present data also suggest that this approach can be further generalized to disambiguate the underpinnings of pharmacological rehabilitation.

## Materials and Methods

### Animals

The heterozygous reeler mouse (B6C3Fe a/a-Relnrl/J strain) breeding pairs were from the Jackson Laboratory. The offspring were genotyped by PCR as previously described^18^. All mice were weaned at 21 days. Mice were housed in standard 12 hours light-dark cycle and supplied food pellets and water *ad libitum*. Ketamine (30 or 100 mg/kg) was administered intraperitoneally prior experiments as previously described^18^. Animals were treated in compliance with the criteria of the European Communities Council Directive (86/609/EEC). Animal experiments were performed under European Union approval (agreement number B 13-055-19).

### Electrophysiology

Coronal prelimbic PFC (PrPFC) slices were prepared as previously described^18^. All experiments were done at 30–32 °C. Picrotoxin (100 μM; Sigma) was added to the artificial cerebrospinal fluid (ACSF) to block GABA_A_ synaptic transmission. Field potentials and whole-cell patch-clamp recordings were made in layer 5, collected using an Axopatch-1D amplifier (Axon Instruments) and acquired with Clampex 10.2 acquisition Software via a Digidata 1440A (Axon Instruments).

For extracellular fields, a stimulating glass electrode filled with ACSF was placed in layer 2/3 as previously described^18^. Field excitatory postsynaptic potentials (fEPSP) were evoked at 0.1 Hz and the glutamatergic nature of the fEPSP was confirmed at the end of each experiment by perfusing the non-NMDA ionotropic glutamate receptor antagonist DNQX (20 μM; Tocris) which specifically blocked the synaptic component without altering the non-synaptic component. LTP was induced using a theta-burst stimulation protocol consisting of five trains of burst with four pulses at 100 Hz, at 200 ms interval, repeated four times at intervals of 10 s. Analysis of both area and amplitude of fEPSP was performed with Clampfit 10.0.0.61 (Axon Instruments). For fEPSP, the non-synaptic component was systematically substracted to fEPSP prior analysis. The magnitude of LTP was calculated 20–30 minutes after TBS as percentage of baseline responses.

Pyramidal neurons in PrPFC layer 5 were visualized using an infrared illuminated upright microscope (Olympus BX51WI). Spontaneous AMPA-EPSCs (AMPA-sEPSCs) were recorded at −70 mV using a potassium-gluconate internal solution^18^. Spontaneous NMDA-EPSCs (NMDA-sEPSCs) were recorded at +40 mV in the presence of DNQX (20 μM) using the following internal solution (mM): cesium-methanesulfonate (143), NaCl (5), MgCl_2_(1), EGTA (1), CaCl_2_ (0.3), Hepes (10), Na_2_ATP (2), NaGTP (0.3) and cAMP (0.2) (pH 7.3 and 290 mOsm). The ratio between AMPA- and NMDA-mediated EPSCs was measured from EPSCs evoked in layer 2/3 while holding layer 5 pyramidal neurons at +40 mV in Cs-methanesulfonate internal solution. The NMDA component of the EPSC was isolated by bath application of NBQX (10 μM; Tocris) and the AMPA-mediated EPSCs were obtained by digital substration of the NMDA-EPSC from the dual component EPSC. Whole-cell recording electrodes had resistances of 4–6 MOhms. Access resistance was continuously monitored (<25 MOhms) and recordings were rejected if there was a >20% change during the course of the experiment. EPSCs were filtered at 2 kHz and digitized at 10 kHz. Spontaneous EPSCs amplitude and inter-interval time were analyzed with Axograph X using a double exponential template: f(t) = exp(-t/rise) + exp(-t/decay). For AMPA-sEPSCs, rise = 0.5 ms and decay = 3 ms, for NMDA-sEPSCs rise = 3 ms and decay = 10 ms. The threshold of amplitude detection was set at 7 pA^18^.

### Dendritic spine reconstruction and quantification

All whole-cell recorded neurons were loaded with neurobiotin through patch pipettes and then processed for post-hoc analysis as described^18^. Only pyramidal neurons from deep layer of the PrPFC showing proper filling of the distal dendritic tree were included in the analysis. Oblique dendrites extending in layer 2/3 were analyzed. Stack images were acquired and analyzed as described^18^.

### Mouse behavior

HRM and littermate controls were trained in 3 phases, fear conditioning (day 1), extinction (days 2 and 3) and renewal training (day 10), using an ABA paradigm as previously described^18^. Fear conditioning was conducted in context A and consisted of 1 min baseline, followed by 5 paired tones (conditioned stimuli, CS) co-terminated with foot shocks (unconditioned stimuli, US). Cue presentations were separated by 1 min. The CS consisted of an 80 db 2.5 kHz tone lasting 20 seconds. The US consisted of 0.5 mA foot shock lasting 1 second. Mice were subjected to two extinction sessions of auditory fear in which each animal was placed in context B during 2 min for baseline monitoring, followed by 12 presentations of the CS on each day. Context-dependent fear renewal was tested seven days after the last extinction session in the conditioning context, i.e. context A using 5 presentations of the CS.

An automated photo sensor-beam activity tracking system was used to measure and score mouse freezing (TruScan 2.0 Colbourn, USA). A freezing event was registered only when inactivity was recorded for a minimum of 2 consecutive seconds. The system was validated against blind manual registration. Results are expressed as a percentage of freezing time of 1 min duration bins.

### Statistical analysis

Univariate multiple comparisons were made using a one-way analysis of variance (ANOVA) followed by a post-hoc Tukey’s Test if significant (GraphPad Prism 5.0). All values are given as mean ± SEM and statistical significance was set at *P* < 0.05.

Multivariate analysis of variance (MANOVA) was used to compare multivariate population means of several groups (age x genotype), thus taking advantage of the multidimensional measurements obtained for each individual. MANOVA based on the multivariate version of the general linear model was performed using Matlab (R2011a).

## Additional Information

**How to cite this article**: Iafrati, J. *et al*. Multivariate synaptic and behavioral profiling reveals new developmental endophenotypes in the prefrontal cortex. *Sci. Rep.* **6**, 35504; doi: 10.1038/srep35504 (2016).

## Supplementary Material

Supplementary Information

## Figures and Tables

**Table 1:** Detailed results of normality test.

Parameters	Genotype	Events >2σ/All Events			
		P10-20	P22-28	P30-45	P50-90
Spine density	wild-type	0/10	**1/9**	0/7	0/8
	HRM	0/13	0/15	0/12	0/6
AMPA-sEPSC	wild-type	0/13	0/9	0/9	0/13
	HRM	0/15	0/13	0/17	0/13
NMDA-sEPSC	wild-type	0/15	0/12	0/8	0/11
	HRM	0/14	0/11	**1/8**	**1/13**
AMPA/NMDA ratio	wild-type	**1/12**	0/8	0/9	0/9
	HRM	0/10	0/8	0/9	0/7
GluN2B	wild-type	0/13	0/7	0/8	0/8
	HRM	0/14	0/8	0/10	0/9
TBS-LTP	wild-type	0/8	0/10	0/11	0/18
	HRM	0/12	0/18	0/13	0/16
Renewal	wild-type	N/A	0/15	**1/14**	0/17
	HRM	N/A	1/21	0/16	**1/17**

The number of points exceeding 2σ per total number of points is indicated for each parameter and condition (genotype, treatment and age). Values in bold indicate where *P* > 0.05.
