# Supplementary material for: Multivariate synaptic and behavioral profiling reveals new developmental endophenotypes in the prefrontal cortex
Source: Sci Rep. 2016 Oct 21;6:35504. doi: 10.1038/srep35504 (PMC5073243; doi:10.1038/srep35504)
Supplement: Supplementary Information [file srep35504-s1.pdf]

Supplementary Information

Multivariate synaptic and behavioral profiling reveals new developmental endophenotypes in the prefrontal cortex.

Jillian Iafrati, Arnaud Malvache, Cecilia Gonzalez Campo, M. Juliana Orejarena, Olivier Lassalle, Lamine Bouamrane and Pascale Chavis.

Supplementary Table 1.

Parameters	Genotype	P10-20	P22-28	P30-45	P50-90	ANOVA
Spine length (μm)	wild-type (n)	1.19±0.04 (10)	1.28±0.03 (11)	1.24±0.03 (7)	1.28±0.06 (8)	$F_{(3,32)}$ =1.277 P=0.2989
	HRM (n)	1.26±0.02 (14)	1.26±0.04 (13)	1.20±0.05 (12)	1.30±0.03 (6)	$F_{(3,41)}$ =0.9568 P=0.4222
Head diameter (μm)	wild-type (n)	0.44±0.02 (10)	0.41±0.01 (11)	0.44±0.02 (7)	0.47±0.01 (8)	$F_{(3,32)}$ =3.089 P=0.0409
	HRM (n)	0.46±0.01 (14)	0.45±0.01 (13)	0.46±0.01 (12)	0.44±0.00 (6)	$F_{(3,41)}$ =1.355 P=0.2700

Summary of the values for spine length and head diameter and statistical tests for each developmental epoch and genotype. Values are expressed as mean ± SEM.

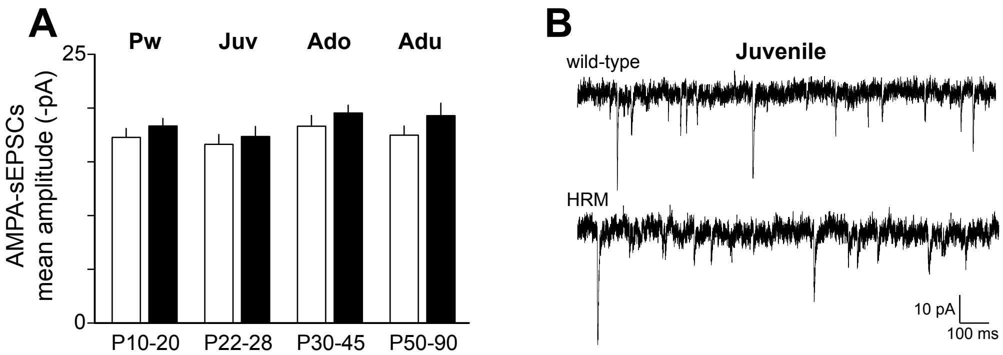

Supplementary Figure 1

Supplementary Figure 1 : Maturational profile of spontaneous AMPA-EPSCs.

(A) Mean amplitude of AMPA-sEPSCs showing no difference between genotypes at all ages tested. Values are at P10-20: 17.3 ± 0.8 pA n=13 wild-type and 18.4 ± 0.7 pA n=15 HRM, at P22-28: 16.6 ± 0.9 pA n=9 wild-type and 17.4 ± 0.9 pA n=13 HRM, at P30-45: 18.3 ±1.0 pA n=9 wild-type and 19.6 ± 0.7 pA n=17 HRM, at P50-90: 17.5 ± 0.9 pA n=13 wild-type and 19.3 ± 1.2 pA n=13 HRM ($F_{(7,94)}$ = 1.337, *P*=0.2415 ANOVA).

(B) Representative traces of AMPA-sEPSCs recorded at -70mV.

A

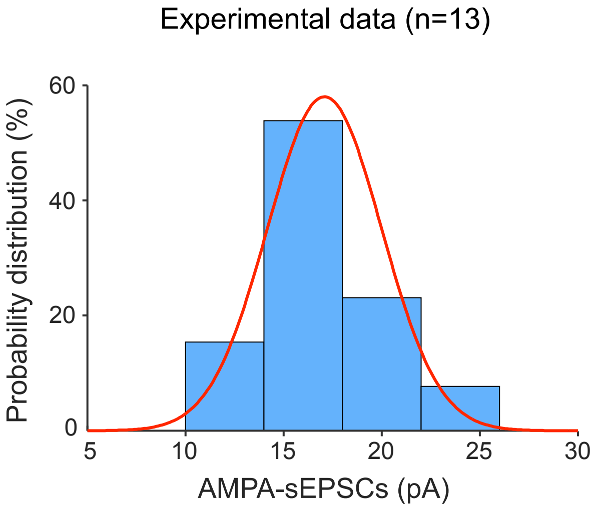

B

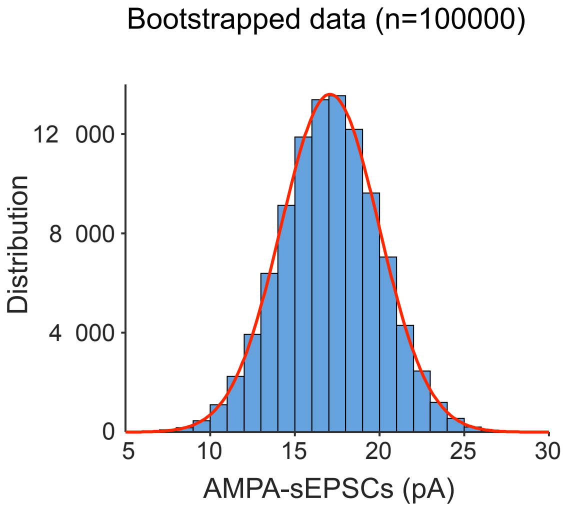

C

Juvenile wild-type virtual mice

Mouse	Spines	AMPA	NMDA	Ratio	GluN2B	LTP	Renew
M1	13.06	14.34	20.01	0.93	64.4	5.78	1.06
M2	15.69	15.09	15.74	1.53	74.84	38.59	148.13
M3	16.44	17.32	20.65	1.08	52.2	25.13	24.84
...	...	...	...	...	...	...	...
M10^5	15.71	14.61	9.00	1.04	68.5	77.11	195.25

D

Juvenile HRM virtual mice

Mouse	Spines	AMPA	NMDA	Ratio	GluN2B	LTP	Renew
M1	12.54	10.97	11.18	0.46	41.8	17.33	43.49
M2	19.52	15.92	22.41	0.64	47.9	-0.02	-34.60
M3	15.19	14.33	6.49	0.92	77.1	-3.15	-60.5
...	...	...	...	...	...	...	...
M10^5	8.23	18.44	18.80	0.06	43.5	2.81	16.49

Supplementary Figure 2

Supplementary Figure 2 : Boostrapping of incomplete datasets.

(A) Distribution of an example parameter, amplitude of AMPA-sEPSCs, in HRM juvenile mice and the corresponding Gaussian fit curve (red).

(B) Distribution of the bootstrapped dataset generated from the fitted experimental distribution shown in A (red curve).

(C-D) Tables showing bootstrapped values obtained for the seven different parameters used for multivariate analysis in virtual juvenile wild-type (C) and HRM (D) mice. The AMPA-sEPSCs amplitude (AMPA column) in HRM mice (D) is sampled in the distribution shown in B.

Spines: Spine density (per 10µm), AMPA: AMPA-sEPSCs amplitude (pA), NMDA : NMDA-sEPSCs amplitude (pA), ratio : AMPA/NMDA ratio, GluN2B : Ro25-6981 sensitivity of evoked NMDA-EPSCs, LTP : percentage of potentiation and renew : percentage of renewal.

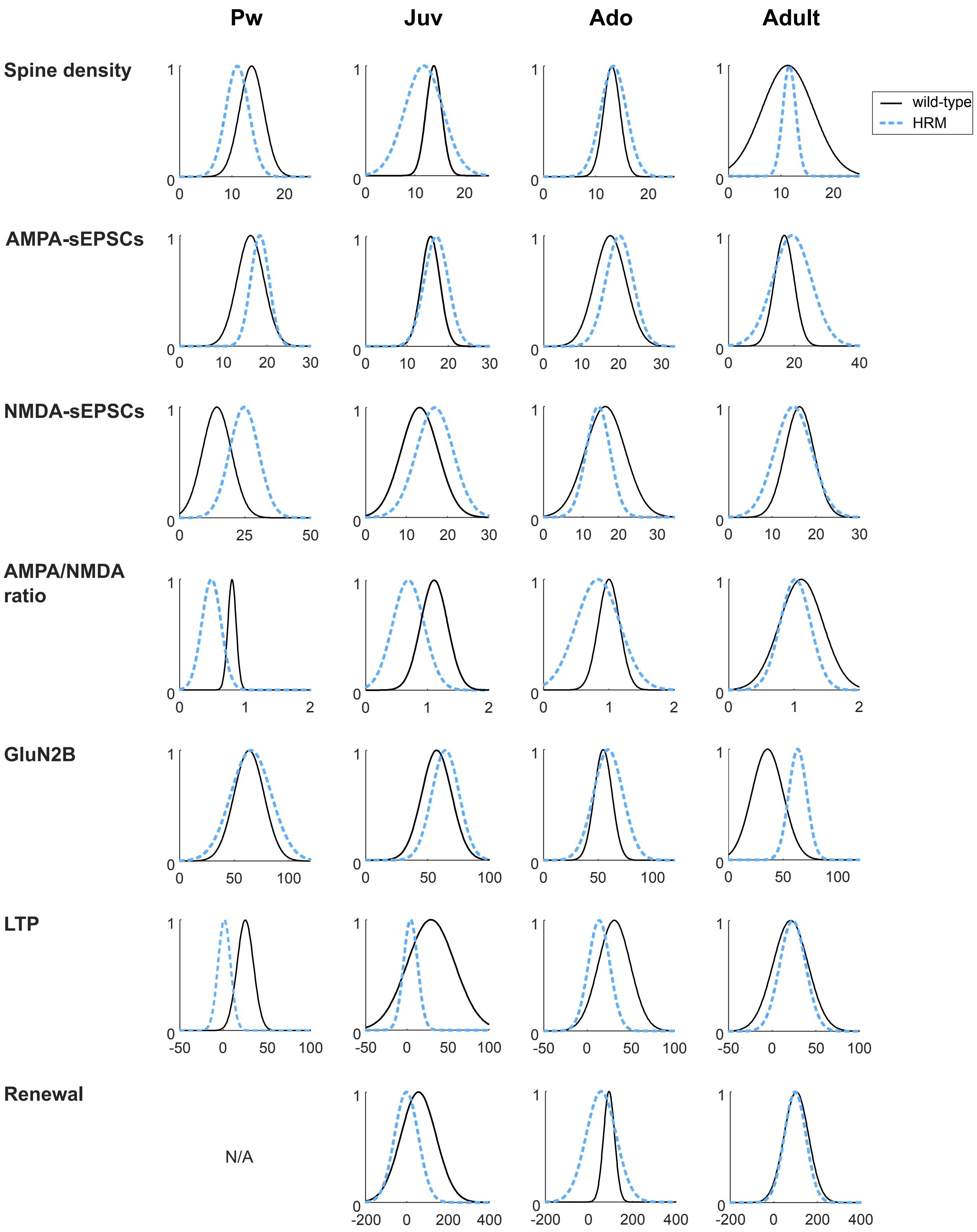

Supplementary Figure 3

Supplementary Figure 3 : Distribution of bootstrapped datasets.

Fitting curves of the distribution of wild-type (black) and HRM (dashed blue) bootstrapped data are depicted for each parameter during maturation. Each graph shows the degree of similarity or difference between wild-type mice and HRM. Curves were normalized to 1. Data presented in the juvenile column (juv) show the fittings of the bootstrapped data presented in Supplementary Figures 2C and D.

Subsequent MANOVA analysis of the bootstrapped values provided for each epoch the weight of the different parameters (see Figure 3A) included in the optimized parameter that maximizes the variance.

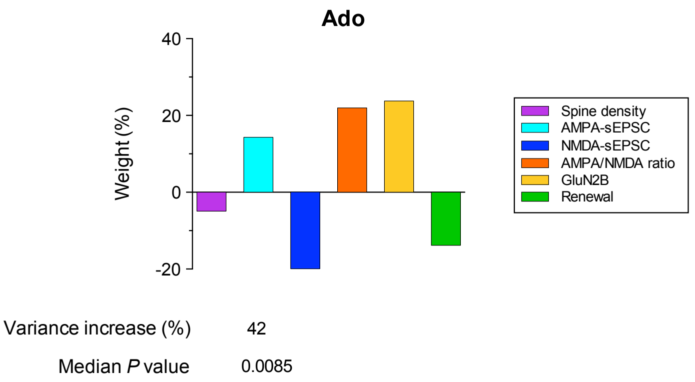

Supplementary Figure 4

Supplementary Figure 4.

Graph depicting the relative weight of the following parameters measured during adolescence: spine density, AMPA- and NMDA-sEPSCs, AMPA/NMDA ratio, Ro25-6981 sensitivity of evoked NMDA-EPSCs (GluN2B) and renewal. The increase in the variance and the median *P* values of the optimized parameter are indicated below.
